# Identification of potential hub genes associated with atopic dermatitis-like recombinant human epidermal model using integrated transcriptomic and proteomic analysis

**DOI:** 10.17305/bb.2023.9439

**Published:** 2024-02-01

**Authors:** Wu Qiao, Tong Xie, Jing Lu, Tinghan Jia, Ken Kaku

**Affiliations:** 1 Pigeon Manufacturing Shanghai CO., LTD., Shanghai, China

**Keywords:** Atopic dermatitis (AD), transcriptomic, proteomic, recombinant human epidermal (RHE), inflammatory

## Abstract

Atopic dermatitis (AD) is a severe inflammatory skin disorder, characterized by elevated levels of proinflammatory cytokines that fuel a vicious cycle of inflammation. While inflammatory recombinant human epidermal (RHE) models relevant to AD have been established, comprehensive understanding remains limited. To illuminate changes and identify potential hub genes involved in AD-related inflammation, RHE models, stimulated by an inflammatory cocktail including polyinosinic-polycytidylic acid, tumor necrosis factor-alpha (TNF-α), interleukin 4 (IL-4), and interleukin 13 (IL-13), were constructed and examined using tandem mass tags-proteomic coupled with RNA-seq transcriptomic analyses. Principal component analysis (PCA), Gene Ontology (GO), and Kyoto Encyclopedia of Genes and Genomes (KEGG) pathway functional enrichment were employed for the analysis of related genes and proteins. Protein–protein interaction networks helped identify hub genes, which were further confirmed by qPCR and western blot. We observed high expression of thymic stromal lymphopoietin in the inflammatory RHE. Our study identified 2369 differentially expressed genes and 880 differentially expressed proteins in the cocktail-induced group versus the normal control group. A total of 248 overlapping symbols were enriched in various biological processes and signaling pathways, including cornification envelope, cell–cell junction, calcium ion binding, extracellular matrix receptor, terpenoid backbone biosynthesis, and peroxisome proliferator-activated receptors signaling pathway, among others. Among the 248 overlapping symbols, CytoHubba identified 10 hub molecules, namely, signal transducer and activator of transcription 3 (STAT3), integrin subunit beta 1 (ITGB1), filaggrin (FLG), involucrin (IVL), DEAD (Asp-Glu-Ala-Asp) box polypeptide 58 (DDX58), small proline-rich protein 1B (SPRR1B), interferon induced with helicase C domain 1 (IFIH1), desmoglein 1 (DSG1), collagen type XVII alpha 1 chain (COL17A1), and integrin subunit alpha 6 (ITGA6), based on the degree. These integrated results offer valuable insights into the molecular mechanisms of AD and present potential tools for screening cosmetic formulations intended for the treatment of AD.

## Introduction

Atopic dermatitis (AD), also known as eczema or atopic eczema, is a common inflammatory disease that affects up to 20% of children and 3% of adults [1, 2]. Unfortunately, the worldwide incidence of AD has increased remarkably and alarmingly, although it is stable in high-income countries. The disease has a heterogeneous clinical presentation among individuals and is characterized by persistent pruritus and recurrent eczematous lesions [3]. Common clinical signs observed in patients with AD include generalized skin dryness, early onset of the disease, and a family history of atopic diseases, such as allergic rhinitis and asthma [4]. The pathophysiology of AD is characterized by a complicated interplay of multiple factors, including a dysfunctional epidermal barrier, abnormalities in the skin microbiome, immunologic dysregulation, inflammation, neuroimmune interactions, and genetic risk factors [5]. Mutations in the filaggrin gene (*FLG*) leading to alterations in the skin barrier protein FLG and profilaggrin represent the best known genetic risk factor associated with abnormalities in lipid chain length [6], water loss, and finally inflammation [7, 8]. AD is classified as a type 2 immune-mediated disease [9]. Cutaneous inflammation plays a key role in the pathogenesis of AD, and overexpression of inflammatory factors is also one of the main features of AD. Lesional AD skin exhibits aberrant expression patterns of proinflammatory factors associated with keratinocytes and T cells, particularly high expression of interleukin 4 (IL-4), interleukin 13 (IL-13), interleukin 4 receptor (IL-4R), interleukin 33 (IL-33), and thymic stromal lymphopoietin (TSLP) [10]. TSLP, a member of the IL-2 cytokine family, is expressed in epidermal cells when the skin barrier is compromised by scratching, antigens (pollen and mites), and cytokines, such as tumor necrosis factor-alpha (TNF-α) [11]. TSLP triggers the activation of dendritic cells (from the human thymic stroma) that upregulate costimulatory molecules, such as CD86, which in turn promotes the proliferation and differentiation of CD4+ T cells and enhances the immune response and the production of IL-4 and IL-13 [12]. In type 2 inflammation, IL-4 and IL-3 play major roles in opening cellular tight junctions [13], reducing structural epidermal proteins such as FLG [14], and defects in endogenous protease inhibitors [15].

Many studies have used tissue engineering and mice to develop in vitro models with the characteristics of AD to investigate disease mechanisms and find potential therapeutic targets [16]. AD mouse models are classified into three groups: 1) inbred models, 2) genetically engineered models, 3) models induced by exogenous agents [17]. Due to The Organization for Economic Cooperation and Development’s promotion of alternative methods to animal testing in cosmetic research, two-dimensional (2D) and three-dimensional (3D) models have become important tools. The simplest model is a 2D cell system such as 2,4-dinitrochlorobenzene (DNCB)-induced AD in HaCaT cells [18]. Recombinant 3D skin constructs can mimic the real skin dermal structures and barrier function. In the context of AD, some recombinant human epidermal (RHE) models have been specifically designed to mimic FLG deficiency [19, 20], while others use TNF-α or polyinosinic-polycytidylic acid (poly I:C) alone or in combination with Th2 cytokines to induce AD-like features in RHE models [21, 22].

Previous studies on AD-related inflammation models have been limited at the transcriptome and morphology levels, and there is no integrated transcriptomic and proteomic study to gain a comprehensive understanding of the AD-like inflammatory model. In view of the above considerations, we prepared the construction of AD-like inflammatory RHE models by adding an inflammatory cocktail (poly I:C, TNF-α, IL-4, and IL-13). Subsequently, RNA-seq and tandem mass tags (TMTs) proteomic technology were performed to examine gene expression and protein profiles of the RHE models under inflammatory and normal conditions (NC). Integrated transcriptomic and proteomic analysis was performed to investigate hub genes. A better understanding of AD-like RHEs will provide deep thinking for further search for mechanization of AD and can be used as a relevant tool for screening cosmetic formulations to alleviate AD.

## Materials and methods

### Cell cultures

Normal human epidermal keratinocytes (NHEKs) were obtained from Guangdong BioCell Biotechnology Co., Ltd. NHEK cells were cultured in a monolayer with epilife growth medium supplemented with human keratinocyte growth supplement (HKGS) (Thermofisher Scientific, Waltham, MA, USA) until they were 80% confluent. Second passage NHEKs were used to generate RHE models.

### Establishing 3D reconstructed human epidermis models

The inserts precoated with collagen I (Corning, NY, USA) were placed in the lowest position in the respective wells. NHEK cells were seeded into the inserts at a density of 3.5 × 105 cells/0.5 cm^2^. The NHEKs were seeded with 0.5 mL epilife growth medium supplemented with HKGS, 10 ng/mL KGF, 140 µM calcium chloride (CaCl_2_), and 50 µg/mL ascorbic acid (Sigma-Aldrich, Burlington, MA, USA) in the lower and upper compartments for two days. The culture was raised to the air–liquid interface and then the appropriate volume of medium was added with an additional 1.5 mM CaCl_2_.

After 16 days in culture, an inflammatory cocktail containing poly I:C 10 µg/mL, TNF-α 10 ng/mL, IL-4 50 ng/mL, and IL-13 50 ng/mL (R&D, Minneapolis, MN, USA) was added to the medium and incubated for 72 h. The 3D RHE models were divided into cocktail-induced positive control RHEs (PC group) and normal control RHEs (NC group) depending on the treatment. These RHE models were used for the following experiments.

### Enzyme-linked immunosorbent assay (ELISA)

The lower compartment medium was collected and stored at −20 ^∘^C. TSLP was quantified using the human TSLP kit (Thermofisher Scientific, Waltham, MA, USA) according to the manufacturer’s instructions.

### RNA extraction and sequencing

RNA extraction and sequencing were performed by TIANGEN Biotech (Beijing) Co, Ltd. RHEs were cut from the inserts with a surgical knife and soaked in trizol (Thermofisher Scientific, Waltham, MA, USA) for RNA extraction. The concentration and purity of the RNA were determined using the Nanodrop 2000 spectrophotometer (Thermofisher Scientific, Waltham, MA, USA), and the integrity of the RNA was analyzed using the Agilent 2100 bioanalyzer.

RNA containing polyA in total RNA was enriched using the TIANSeq mRNA Capture Kit (TIANGEN, Beijing, China). TIANSeq Fast RNA Library was used to construct the transcriptome sequencing libraries sequenced on Illumina Novaseq 6000 platform, obtaining 150 bp paired-end reads. HISAT2 [23] was used to map the clean reads obtained from the raw files in fast format to the reference genome. The fragments per kilobase of the exon model per million mapped fragments (FPKM) were calculated using the featureCounts software [24] for subsequent analysis. The detailed versions of the software used in the study are listed in Table S1. RNA-seq data are available through NCBI with the identifier SRR24941377-82. 

### Protein preparation and tandem mass tag quantification

RHE samples were quickly frozen in liquid nitrogen, placed in PASP lysis buffer (100 mM NH4HCO3, 8 M urea, pH 8), and ultrasonicated on ice for 5 min. The lysate was centrifuged at 12000 *g* for 15 min at 4 ^∘^C and supernatant was added 10 mM DTT for 1 h followed by alkylation with IAM for 1 h in the dark. Samples were mixed with 4 times the volume of cooled acetone and incubated overnight at −20 ^∘^C, then centrifuged at 12000 *g* for 15 min at 4 ^∘^C and the precipitate was collected, which was dissolved in dissolution buffer (8M urea, 100 mM TEAB, pH 8.5). The amount of sample solution was measured by the Bradford method and the quality of protein was determined by 12% SDS-PAGE.

The dissolved protein samples were collected and the volume was made up to 100 µL with dissolution buffer. The sample was mixed with trypsin and 100 mM TEAB buffer and digested overnight at 37 ^∘^C. Labeling samples were desalted and lyophilized after being treated with acetonitrile-dissolved TMT labeling reagent. A C18 column (Waters BEH C18, 4.6 × 250 mm, 5 µm) on a Rigol L3000 HPLC system was used to separate the fractions. Finally, fractions were analyzed using the EASY-nLC 1200 UHPLC system coupled to the Q Exactive HF-X mass spectrometer operating in data-dependent acquisition (DDA) mode. Proteome Discoverer 2.4 was used to identify and quantify the peptides and proteins. Mass spectrometry proteomics data were deposited with the ProteomeXchange Consortium via the partner repository PRIDE [25] with dataset identifier PXD043085.

### Identification of differentially expression genes (DEGs) and proteins (DEPs)

The DESeq2 package was used to identify DEGs with criteria |log2Foldchange|>2 and adjusted *P* value < 0.05. DEPs were analyzed with a score of logFoldchange > 1.2 or logFoldchange < 0.83 and false discovery rate < 0.05. All DEGs and DEPs were performed using normal RHEs as reference.

### Bioinformatic analysis

Principal component analysis (PCA) was performed using the Factoextra R packages with FPKM values for RNA-Seq and mass spectrometry intensities for TMT. The clusterProfier R package was used for gene enrichment analysis of gene ontology (GO) and Kyoto Encyclopedia of Genes and Genomes (KEGG) pathways based on the org.Hs.eg.db database. Metascape (https://metascape.org/) was used for the analysis of protein GO and KEGG. Heatmap was created using complexHeatmap and Circlize packages, while other plots were created using ggplot2. The detailed versions of the packages used in the study are listed in Table S2.

### Protein–protein interaction (PPI) network and hub gene analysis

A PPI network of selected symbols was plotted using the Search Tool for the Retrieval of Interacting Genes (STRING) (http://string-db.org) to assess all protein interaction relationships. The PPI network was visualized using Cytoscape software (version 3.9.2). The Cytohubba Apps plugin was used to analyze the hub genes that play key roles in the PPI network by calculating the hubba top 10 degree nodes. The PPI network graph was drawn and optimized using Cytoscope.

### Quantitative real-time PCR (qRT-PCR)

Relative mRNA expression of specific genes was determined by qRT-PCR. Total RNA was isolated using Trizol (Invitrogen, Carlsbad, CA, USA) according to the manufacturer’s instructions. Isolated RNA was reverse transcribed into cDNA using the FastKing cDNA RT kit (TIANGEN, Beijing, China), and aliquots were stored at −20 ^∘^C. Quantitative PCR was performed using commercially available reagents and the LC 96 qPCR system with specific primers (Table S3). The results obtained were normalized to the level of the glyceraldehyde-3-phosphate dehydrogenase (*GADPH*) gene, and the relative expression of the gene was calculated by 2 ^−ΔΔCt^.

### Western blot

Protein extraction from RHEs was performed using the same method as described in the proteomic protocol above. Protein concentration was determined using the BCA assay. Protein samples were separated on 10% tris-glycine gels using SDS-PAGE and transferred to a PVDF membrane using the power blotter system (Thermofisher Scientific, Waltham, MA, USA). The PVDF membrane was blocked with 5% BSA, washed with PBST, and coated with the primary antibody including FLG (NPB1-87528, 1:2000, Novus), involucrin (IVL) (NBP2-16981, 1:2000, Novus), aquaporin (AQP3) (PA5-78811, 1:2000, Thermofisher), signal transducer and activator of transcription 3 (STAT3) (9139, 1:1000, CST), phospho-STAT3 (9145, 1:1000, CST), and β-actin (3700, 1:1000, CST). HRP goat anti-rabbit secondary antibody (ab6721, 1:10000, Abcam) was used and incubated for 1 h at room temperature. The membrane was exposed to enhanced chemiluminescence (ECL) reagents (AP34L024, Life-iLab Biotech, Shanghai, China) and imaged using the iBright FL1000 Imaging System (Thermofisher Scientific, Waltham, MA, USA).

### Statistical analysis

Differences between the means of variables containing ELISA, qPCR, and western blot measured in triplicates of normal and cocktail-induced RHE models were tested with a student’s *t*-test using GraphPad Prism 9.0. *P* < 0.05 was considered statistically significant in each experiment.

## Results

### Compromised reconstructed epidermis and ELISA assay

**Figure 1.**
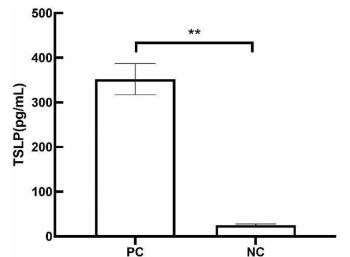
**The ELISA results of TSLP.** **P < 0.01. TSLP: Thymic stromal lymphopoietin; PC: Inflammatory cocktail (positive control) group; NC: Normal control group.

To mimic the inflammatory milieu observed in AD, we selected four factors (poly I:C, TNF-α, IL-4, and IL-13) as the inflammatory cocktail according to previous literature [21]. Our previous results of hematoxylin-eosin (HE) stain indicated that the living layers of the cocktail-induced PC group were looser and showed spongiosis compared with normal RHEs, NC group [26]. To confirm the relationship between damaged RHEs and the inflammatory content observed in AD patients, we performed ELISA analysis to quantify the secretion of TSLP. As shown in Figure 1, the inflammatory cocktail significantly increased the level of TSLP in the PC group compared with the NC group (*P* < 0.01).

### Sample distribution and correlation

The RNA sequencing depth of this study was approximately 6G with three replicates per group. The total quantification of peptides in TMT proteomic analysis was 46,530. The violin plot and box plot showed the distribution of gene and protein expression using FPKM values and relative quantitative peptide spectrum matches (PSM) (Figures S1 and S2). The correlation coefficients of FPKM values and quantitative PSM values in each sample were calculated. The similarities between different groups were visualized using the pheatmap and circlize packages. As shown in Figures S1 and S2, the RHEs exhibited excellent biological duplication under rooster tail-induced (PC group) and NC group, indicating the high reliability of the experimental design.

### PCA analysis of recombinant human epidermal (RHE) samples

**Figure 2.**
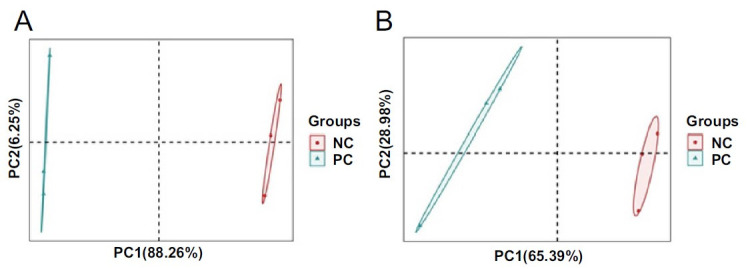
**Principal component analysis (PCA) plot.** (A) PCA plot of transcriptomic analysis; (B) PCA plot of proteomic analysis. PC: Inflammatory cocktail (positive control) group; NC: Normal control group.

PCA was used to analyze the differences between the six RHE samples. Based on genes and proteins, the distribution plots of PCA for the different samples were shown in Figure 2A and 2B, respectively. The variance contributions of the first principal components were 88.26% and 65.39% at the gene and protein levels, respectively. The RHEs were grouped in two separate clusters in both graphs, which clearly separated the NC group from the PC group and also showed a clear grouping of biological triplicates. This result suggests that the cocktail-induced RHEs have significant differences in terms of gene and protein expression.

### Analysis of transcriptomic sequencing expression

**Figure 3.**
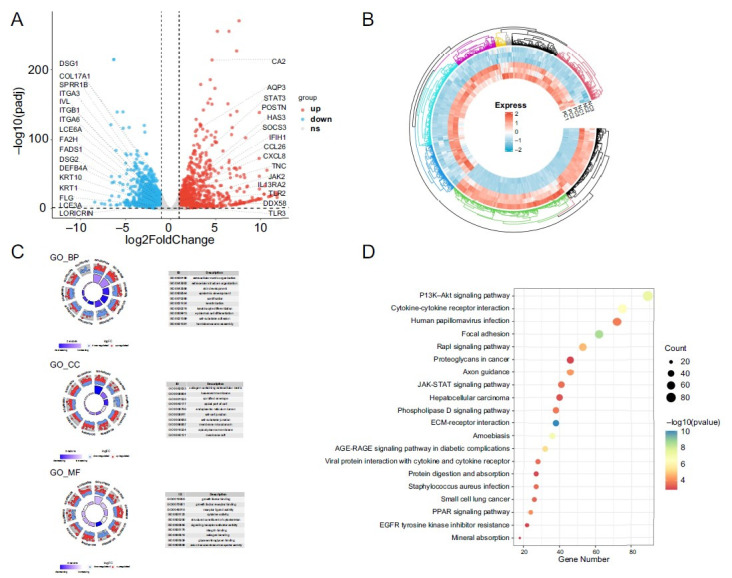
**Transcriptomic analysis of recombinant human epidermalmodels under inflammatory cocktail positive control group and normal control group.** (A) Volcano plot of differential gene analysis; (B) Circlize heatmap plot of differential gene analysis; (C) Top 30 enriched GO terms of differential gene analysis; (D) Bubble plot of the top 20 KEGG pathways. GO: Gene Ontology; KEGG: Kyoto Encyclopedia of Genes and Genomes; JAK-STAT: Janus kinases signal transducer and activator of transcription; ECM: Extracellular matrix; PPAR: Peroxisome proliferator-activated receptor; PI3K-Akt: Phosphatidylinositol 3-kinase protein kinase B.

Based on the threshold criteria of |log2foldchange|>1 and padj < 0.05, we identified 2963 DEGs from the screening of 13,482 genes in this study, with 1178 upregulated genes and 1785 downregulated genes (Figure 3A). Some representative genes, such as *FLG*, *IVL*, loricrin (*LOR)*, *AQP3*, and keratin 10 (*KRT10)* were labeled. The heatmap diagram of DEGs showed optimal clustering and well-controlled biological replications among the PC group and NC group (Figure 3B). As shown in Figure 3C, the 30 terms of GO were selected to create a GOcircle plot of the biological process (BP), molecular function (MF), and cell component (CC). At BP, DEGs were predominantly associated with extracellular matrix (ECM) organization, extracellular structure organization, skin development, and epidermis development. At MF, DEGs were predominantly associated with growth factor binding, growth factor receptor binding, and receptor ligand activity. At CC, DEGs were predominantly associated with collagen-containing ECM, basement membrane, and cornified envelope. For KEGG assessment of DEGs, the clusterProfile package was used, and the top 20 KEGG pathways were selected based on their importance and relevance to the study (Figure 3D). The phosphatidylinositol 3-kinase protein kinase B (PI3K-Akt) pathway, cytokine–cytokine receptor interaction, focal adhesion, Janus kinases signal transducer and activator of transcription proteins (JAK-STAT), peroxisome proliferator-activated receptors (PPAR) pathway, and ECM–receptor interaction were enriched at the gene level.

### Differential analysis of protein expression

**Figure 4.**
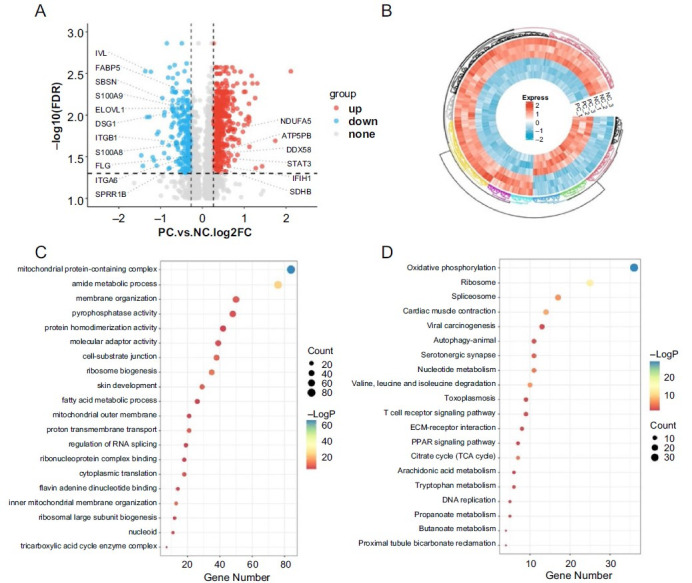
**Proteomic analysis of recombinant human epidermalmodels under inflammatory cocktail positive control group and normal control group.** (A) Volcano plot of differential protein analysis; (B) Circlize heatmap plot of differential protein analysis; (C) Top 20 enriched GO terms of differential protein analysis; (D) Bubble plot of the top 20 KEGG pathways. GO: Gene Ontology; KEGG: Kyoto Encyclopedia of Genes and Genomes; IVL: Involucrin; FABP5: Fatty acid-binding protein 5; SBSN: Suprabasin; S100A9: S100 Calcium binding protein A9; ELOVL1: ELOVL fatty acid elongase 1; DSG1: Desmoglein 1; ITGB1: Integrin subunit beta 1; S100A8: S100 calcium binding protein A8; FLG: Filaggrin; ITGA6: Integrin subunit alpha 6; SPRR1B: Small proline-rich protein 1B; NDUFA5: NADH:Ubiquinone oxidoreductase subunit A5; ATP5PB: ATP synthase peripheral stalk-membrane subunit B; DDX58: DEAD (Asp-Glu-Ala-Asp) box polypeptide 58; STAT3: Signal transducer and activator of transcription 3; IFIH1: Interferon induced with helicase C domain 1; SDHB: Succinate dehydrogenase complex iron sulfur subunit B; ECM: Extracellular matrix; PPAR: Peroxisome proliferator-activated receptor.

After scanning the protein database, we identified a total of 4673 proteins, including 643 upregulated proteins and 237 downregulated proteins, using fold-change > 1.2 or fold-change < 0.83 and false discovery rate < 0.05 as screening standards. The volcano and heatmap plot showed the different distribution of DEPs in Figure 4A and 4B. Some representative proteins, such as FLG, IVL, AQP3, and S100 calcium-binding protein A9, had been labeled. The top 20 terms of GO showed that DEPs were enriched in mitochondrial protein-containing complex, amide metabolic process, membrane organization, cell-substrate compound, fatty acid metabolic process, and skin development, as shown in Figure 4C. The dot plot of KEGG showed that DEPs were mainly enriched in oxidative phosphorylation, ribosome, nucleotide metabolism, T-cell receptor signaling pathway, ECM–receptor interaction, PPAR signaling pathway, and tricarboxylic acid (TCA) cycle (Figure 4D).

### Integrated transcriptomic and proteomic analysis

**Figure 5.**
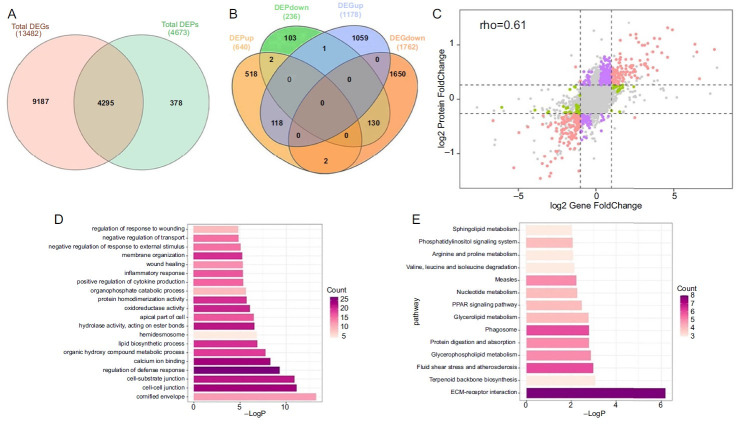
**Integrated transcriptomic and proteomic analysis.** (A) Venn diagram showing the overlap of total genes and proteins; (B) Venn diagram showing the overlap of DEGs and DEPs; (C) Nine quadrant diagrams showing the change trend of DEGs and DEPs; (D) Bar plot of the top 20 GO terms; (E) Bar plot of the top 14 KEGG pathways. DEG: Differentially expressed genes; DEP: Differentially expressed proteins; GO: Gene ontology; KEGG: Kyoto Encyclopedia of Genes and Genomes; ECM: Extracellular matrix; rho: The correlation coefficient between log2 gene foldchange and log2 protein foldchange.

We combined the results of transcriptomic and proteomic data to gain a deeper understanding of the AD in RHE models. Compared with the normal RHEs, a total of 13,482 genes and 4673 proteins were identified in the PC groups by the differential expression analysis of transcriptome and proteome, of which 4295 factors were found to be regulated at both the RNA and protein levels (Figure 5A). Of the 4295 factors, 248 factors were consistent at both the RNA and protein levels, including 118 upregulated and 130 downregulated. At the protein level, there were 520 upregulated and 103 downregulated, and at the RNA level, there were 1059 upregulated and 1650 downregulated (Figure 5B). As shown in Figure 5C, the Pearson correlation coefficient of the log2 fold-change values at the protein and gene levels was 0.61, indicating a high correlation trend at the mRNA level and the corresponding protein level. There were multiple differences (log2 values) at different levels, showed in nine quadrant diagrams (Figure 5C). Red dots indicated the same trend in upregulation and downregulation of proteins/genes. The presence of purple dots indicated differential changes at the protein level without corresponding changes at the gene level. The green dots indicated differential changes at the gene level without corresponding changes at the protein level. The gray dots represent situations in which no significant difference was observed at both the protein and gene levels. In the results of the integrated transcriptomic and proteomic analysis results, the top 20 terms, including BP, CC, and MF, were shown in Figure 5D. The genes and proteins with a similar trend were enriched in the cornified envelope, cell–cell junction, cell–substrate junction, defense response regulation, calcium ion binding, organic hydroxy compound metabolic process, and lipid biosynthesis process. After KEGG pathway enrichment analysis and annotation with the Metascope database, the top eight pathways were identified from the results of proteomic and transcriptomic analysis (Figure 5E). ECM–receptor interaction, terpenoid backbone biosynthesis, fluid shear stress and atherosclerosis, glycerophospholipid metabolism, glycerolipid metabolism, PPAR signaling pathway, and amino acid metabolism were enriched with genes showing the same expression trends in the proteome and transcriptome.

### PPI network and hub gene identification

**Figure 6.**
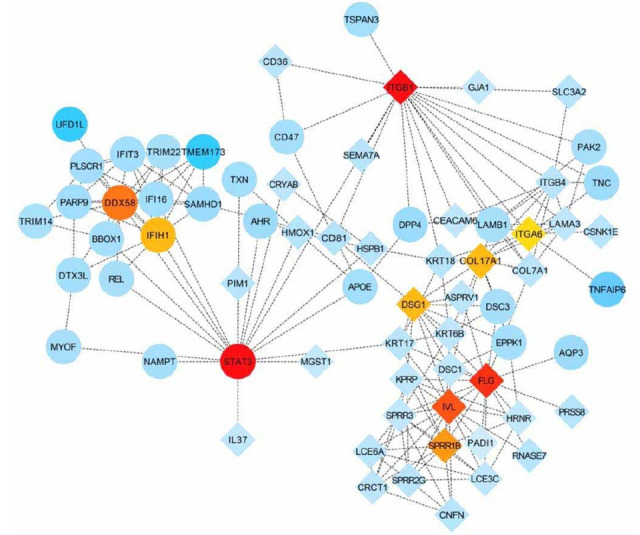
**PPI network and hub genes.** Circles represent upregulation and diamonds represent downregulation of genes and proteins. Genes are differentiated in blue based on their log2foldchange values. PPI: Protein–protein interaction.

The PPI network with overlapping symbols containing 118 common upregulated genes and 130 common downregulated genes was generated from the STRING database. The hub genes from the PPI network were analyzed using the degree algorithm in the CytoHubba app plugin, and the top 10 hub genes were ranked based on the degree scores (Table 1), including signal *STAT3*, integrin subunit beta 1 (*ITGB1)*, *FLG*, *IVL*, DEAD (Asp-Glu-Ala-Asp) box polypeptide 58 (*DDX58)*, small proline-rich protein 1B (*SPRR1B)*, interferon induced with helicase C domain 1 (*IFIH1)*, desmoglein 1 (*DSG1)*, collagen type XVII alpha 1 chain (*COL17A1)*, and integrin subunit alpha 6 (*ITGA6)*. As shown in Figure 6, the top ten hub genes have been highlighted in red according to their degree ranking (Table 1). Other genes are distinguished based on their log2foldchange values, with circles representing upregulation and diamonds representing downregulation of genes and proteins.

**Table 1:** Top 10 genes in network ranked by degree method

**Rank**	**Name**	**Score**
1	*STAT3*	18
1	*ITGB1*	18
3	*FLG*	16
4	*IVL*	15
5	*DDX58*	14
6	*SPRR1B*	12
7	*IFIH1*	11
7	*DSG1*	11
7	*COL17A1*	11
10	*ITGA6*	10

STAT3: Signal transducer and activator of transcription 3. ITGB1: Integrin subunit beta 1; FLG: Filaggrin; IVL: Involucrin; DDX58: DEAD (Asp-Glu-Ala-Asp) box polypeptide 58; SPRR1B: Small proline-rich protein 1B; IFIH1: Interferon induced with helicase C domain 1; DSG1: Desmoglein 1; COL17A1: Collagen type XVII alpha 1 chain; ITGA6: Integrin subunit alpha 6.

### Verification of gene and protein expression

**Figure 7.**
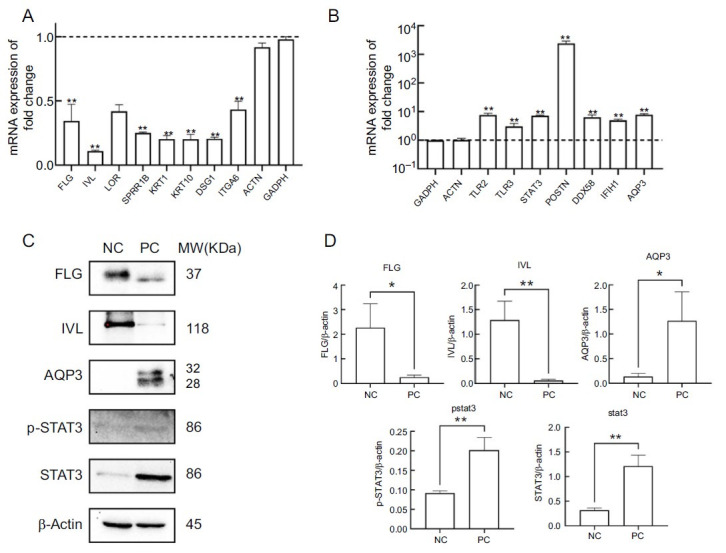
**Verification of relevant genes and proteins.** *P < 0.05; **P < 0.01. (A) The results of downregulation genes between PC group and NC group (n ═ 3); (B) The results of upregulation genes between PC group and NC group; (C) Expression of p-STAT3, STAT3, FLG, IVL, AQP3 and β-actin protein in RHE models (β-actin as control); (D) Statistical data of expression of p-STAT3, STAT3, FLG, IVL and AQP3 protein in each group. WB: Western blot; PC: Inflammatory cocktail positive control group; NC: Normal control group; p-STAT3: P-signal transducer and activator of transcription 3; FLG: Filaggrin; IVL: Involucrin; SPRR1B: Small proline rich protein 1B; KRT1: Keratin 1; KRT10: Keratin 10; DSG1: Desmoglein 1; ITGA6: Integrin subunit alpha 6; ACTN: Actinin; GADPH: Glyceraldehyde-3-phosphate dehydrogenase; TLR2: Toll like receptor 2; TLR3: Toll like receptor 3; STAT3: Signal transducer and activator of transcription 3; POSTIN: Periostin; DDX58: DEAD (Asp-Glu-Ala-Asp) box polypeptide 58; IFIH1: Interferon induced with helicase C domain 1; AQP3: Aquaporin.

To further validate the accuracy of the omics-related data and the above results, we then used qPCR and western blot methods to detect the expression of relevant genes and proteins under inflammatory cocktail conditions and under NC. The expression of 13 genes was validated by qPCR. As shown in Figure 7A, the expression of differentiation-associated genes *IVL, LOR, FLG*, keratin 1 (*KRT1*), *KRT10, SPRR1B*, and *DSG1* was significantly downregulated (*P* < 0.01). The expression of toll-like receptor 2 (*TLR2*), *TLR3*, *STAT3,* and *IFIH1* genes associated with inflammation and immunity was significantly upregulated (Figure 7B). In addition, other epidermal-related genes *AQP3*, periostin (*POSTN*), and *DDX58* were also significantly upregulated (*P* < 0.01). The observed changes in these genes were consistent with the transcriptomic results, which are consistent with previous reports in AD patients. Compared with the NC group, protein expression of FLG (*P* < 0.05) and IVL (*P* < 0.01) was decreased in the positive control group of RHE models. Compared with the NC group, the protein expression of AQP3 (*P* < 0.05), STAT3, and p-STAT3 (*P* < 0.01) was significantly increased (Figure 7C and 7D).

## Discussion

Replication in vitro of the relative gene expression changes associated with the pathogenesis of AD is a major challenge, mainly because of the complicated interplay of the immune system and environmental factors in individuals. However, the reproducibility and convenience of in vitro models are irreplaceable while maintaining a high degree of representativeness. It is critical to recognize the limitations of RHE models in accurately reproducing the complex immunological response.

First, in the current experiment, we generated AD-like RHE models by adding the inflammatory cocktail (IL-3, IL-4, TNF-α, and poly I:C) to the culture medium for 72 h. The inflammatory factors and their respective concentrations were obtained from the literature [21], and the stimulation duration was based on our previous study [26]. The non-lesional AD skin exhibited histological changes resembling spongiosis, consistent with the results of HE staining. Ohtani et al. found that upregulation of HAS3 mRNA stimulated with IL-4/IL-13 in keratinocytes caused spongiosis in acute eczema [27]. In the transcriptomic results, the mRNA expression of HAS3 was also significantly upregulated, which is consistent with the literature. In the lesional skin of AD, dysregulated expression of a wide range of genes primarily related to keratinocyte activity and T-cell infiltration was observed. In particular, genes associated with Th2 response (IL-4, IL-10, and IL-13) and Th22 response (IL-22) showed prominent alterations in their expression levels [28, 29]. TSLP was strongly expressed by keratinocytes in AD patients with skin lesions. The combination of Th2- and TNF-α-induced TSLP production in full-thickness human skin explants, whereas TNF-α alone had no significant effect on TSLP levels [30]. Poly I:C as an agonist of TLR3 ligand mimicking viral double-stranded RNA (dsRNA) could increase TSLP concentration, which could directly activate dendritic cells to secrete Th2-recruiting chemokines [31]. The ELISA result of TSLP showed that the combined inflammatory cocktail effectively stimulated secretion.

Secondly, it should be noted that RHEs, being derived solely from keratinocytes differentiation, lack immune system and the ability to distinguish the skin lesional and non-lesional states. The result of volcano showed that downregulation of *FLG, IVL, ITGB1, ITGA3, KRT10, KRT1, LOR, DSG1*, and *DSG2*, and upregulation of carbonic anhydrase 2 (*CA2*), C-C motif chemokine ligand 26 (*CCL26*), *TLR2*, suppressor of cytokine signaling 3 (*SOCS3*), tenascin-C (*TNC*), and *AQP3*. These genes have been associated with skin barrier function and signal transduction [32]. GO functional classification annotation showed that the DEGs were enriched in ECM, structural organization, skin development, growth factor, and receptor binding (Figure 3C). Interestingly, Christian Cole observed GO enrichment in the areas of extracellular space, receptor binding, and defense response. He compared 26 AD cases with ten control cases [7]. Cytokine–cytokine receptor interaction signaling molecules and interaction and signatures associated with diseases such as measles. Downregulated DEGs were enriched in KEGG pathway signaling pathways, including ECM–receptor interaction, focal adhesion cellular community, and PPAR signaling pathway (Figure S3) [33, 34].

Third, the correlation between mRNA and corresponding protein expression levels depends on various regulatory factors and metabolic processes [35]. The volcano proteomics result also showed that the presence of the inflammatory cocktail caused a delay in the synthesis of barrier proteins, including FLG, IVL, and LOR, which was also observed in other AD-like models and in individuals with AD [36, 37]. Suprabasin (SBSN), a novel precursor of the epidermal differentiation envelope precursor, showed low expression of SBSN in the stratum corneum of AD patients. Masahiro discovered that there was no effect of IL4/IL-13 on SBSN expression in RHE, but our proteomic data showed downregulation of SBSN, although there was no difference at the mRNA level. This discrepancy could be attributed to the influence of TNF-α and poly I:C [38]. The GO and KEGG of DEPs were performed using the Metascape database. It was found that the downregulated DEPs were enriched in skin development, lipid biosynthetic biosynthesis process, cell–cell adhesion, and desmosome desmosomes (Figure S4), which are considered important features of AD [39]. In contrast to the transcriptomes, the upregulated DEPs were enriched in the GO terms mitochondrial protein-containing complex, mitochondrial matrix, and peptide biosynthetic process. 

Leman et al. found that mitochondrial activity is upregulated in non-lesional AD (NLAD). NLAD keratinocytes exhibited increased mitochondrial oxidation of long-chain fatty acids, and metabolomic analysis revealed upregulation of TCA cycle turnover [40]. Interestingly, the upregulated pathways identified in KEGG pathway analysis, such as oxidative phosphorylation, ribosome, citrate cycle (TCA cycle), and amino acid degradation, are consistent with Geraldine, further supporting our findings. PPARs were a group of nuclear hormone receptors that can be divided into three subclasses: PPARα, PPARβ/δ, and PPARγ [41]. Studies had demonstrated the beneficial effects of PPARα agonists on the maintenance of barrier homeostasis including the upregulation of molecules associated with epidermal differentiation and lipid synthesis [42].

Finally, the integrated analysis of DEPs and DEGs identified 248 overlapping genes that were enriched in GO terms, including cornified envelope, cell-substrate junction [37], regulation of defense response, and calcium ion binding [43]. The KEGG pathway contains ECM–receptor interaction [44], Terpenoid backbone biosynthesis, and lipid metabolism. The elongase of very long chainfatty acid 1 (ELOVL1) was involved in the biosynthesis of long-chain free fatty acids (FFAs). Interestingly, there was a discrepancy between the mRNA and protein expression of ELOVL1, as the mRNA showed no significant changes while the protein was downregulated [22]. In AD, AQP3 is upregulated to maintain barrier stability when FLG expression is low (Figure 7).

The top 10 hub gene include *STAT3, ITGB1, FLG, IVL, DDX58, SPRR1B, IFIH1, DSG1, COL17A1*, and *ITGA6*. IL-13/ IL-4 signaling is now considered to be the essential core of the pathogenesis of AD [16, 32]. The cytokines IL-13 and IL-4 bind to the IL-4Rα/ IL-13Rα1 receptor, triggering downstream JAK1/JAK2 and STAT6/STAT3. Activation of the STAT6/STAT3 axis leads to downregulation of FLG expression, disruption of the skin barrier function, and an increase in TSLP production [45]. In the epidermal keratinocytes of individuals with AD, IL-4 binds to its receptors, IL-4α, and triggers activation of STAT3. This activation subsequently stimulates the skin inflammation and contributes to the progression of AD [46]. In this study, both STAT3 and p-STAT3 showed the significant upregulation induced with triggered by the inflammatory cocktail, which in turn led to downregulation of FLG and IVL, as well as upregulation of AQP3 (Figure 7). Surprisingly, IL-13 did not significantly affect the expression levels of IL-4R and IL-13Rα1 in keratinocytes, but it induced the upregulation of IL-13Rα2 in cells exposed to IL-13 [47].

Under physiological conditions in AD, the homeostasis of skin barrier function depends on the precise expression of barrier-related proteins, intercellular lipids, and desmosomes within the granular and keratinized layers of the epidermis [48]. FLG and IVL play key roles in the skin barrier. SPRR proteins have been identified as crucial components of the cornified envelope, serving as cross-bridging that connect LOR [49]. In our experiment, the addition of poly I:C resulted in the upregulation of DDX58 and interferon induced with IFIH1 [50], both of which are dsRNA sensors and play a critical role in the immunological function of the epidermal barrier. Corneodesmosomes play a central role in maintaining the structural integrity of the stratum corneum, while desmosomal cadherins function as intercellular adhesive junctions that connect keratinocytes through their association with intermediate filaments. Research suggests that IL-4 and IL-13 downregulate DSG1 expression in both the cultured human keratinocyte (HaCaT) cell line and the skin of people with AD, which may contribute to the impaired skin barrier observed in individuals with AD [51]. In patients with AD, ITGA6 shows marked expression on endothelial cells in both lesional and non-lesional skin [52]. It is interesting to observe that inflammatory differentiated keratinocytes show a remarkable decrease in the expression of both undifferentiated markers, tumor protein P63 (TP63) and ITGA6, and differentiated markers, including KRT1 and KRT10, at the transcriptional level [53]. COL17A1 is a protein involved in the formation of hemidesmosomes, which are structures that anchor the epidermis. Understanding the role of COL17A1 in AD may have implications for the development of targeted therapies aimed at restoring the integrity of the epidermal barrier [54].

The comprehensive integration of IL-4, IL-13, TNF-α, and ploy I:C into the RHE model enabled a multidimensional simulation of the immunological aspects of AD, which includes TLR2-, TLR3- and TLR4-mediated inflammation. The integrated results provided a deeper understanding of the RHE inflammation model and offered valuable insights for further research and practical applications.

## Conclusion

This study provided comprehensive transcriptome and protein profiling in RHE models that mimic the inflammation associated with AD. Integrated transcriptomic and proteomic analysis further revealed alterations in signaling pathways and potential hub genes. These results provide valuable insights into the molecular mechanisms of AD as well as potential biomarkers for screening cosmetic formulations for the treatment of AD.

## Supplemental data

Supplementary data can be found at the following link: https://www.bjbms.org/ojs/index.php/bjbms/article/view/9439/2914

## References

[1] Totri CR, Diaz L, Eichenfield LF (2014). 2014 update on atopic dermatitis in children. Curr Opin Pediatr.

[2] Silverberg JI, Hanifin JM (2013). Adult eczema prevalence and associations with asthma and other health and demographic factors: a US population-based study. J Allergy Clin Immunol.

[3] Langan SM, Irvine AD, Weidinger S (2020). Atopic dermatitis. Lancet.

[4] Kulthanan K, Tuchinda P, Nitiyarom R, Chunharas A, Chantaphakul H, Aunhachoke K (2021). Clinical practice guidelines for the diagnosis and management of atopic dermatitis. Asian Pac J Allergy Immunol.

[5] Apfelbacher CJ, Diepgen TL, Schmitt J (2011). Determinants of eczema: population-based cross-sectional study in Germany. Allergy.

[6] van Smeden J, Janssens M, Kaye EC, Caspers PJ, Lavrijsen AP, Vreeken RJ (2014). The importance of free fatty acid chain length for the skin barrier function in atopic eczema patients. Exp Dermatol.

[7] Cole C, Kroboth K, Schurch NJ, Sandilands A, Sherstnev A, O’Regan GM (2014). Filaggrin-stratified transcriptomic analysis of pediatric skin identifies mechanistic pathways in patients with atopic dermatitis. J Allergy Clin Immunol.

[8] Dang NN, Pang SG, Song HY, An LG, Ma XL (2015). Filaggrin silencing by shRNA directly impairs the skin barrier function of normal human epidermal keratinocytes and then induces an immune response. Braz J Med Biol Res.

[9] Muraro A, Lemanske RF, Hellings PW, Akdis CA, Bieber T, Casale TB (2016). Precision medicine in patients with allergic diseases: airway diseases and atopic dermatitis-PRACTALL document of the European Academy of Allergy and Clinical Immunology and the American Academy of Allergy, Asthma & Immunology. J Allergy Clin Immunol.

[10] Czarnowicki T, Krueger JG, Guttman-Yassky E (2017). Novel concepts of prevention and treatment of atopic dermatitis through barrier and immune manipulations with implications for the atopic march. J Allergy Clin Immunol.

[11] Lee HC, Ziegler SF (2007). Inducible expression of the proallergic cytokine thymic stromal lymphopoietin in airway epithelial cells is controlled by NFkappaB. Proc Natl Acad Sci U S A.

[12] Nakajima S, Igyártó BZ, Honda T, Egawa G, Otsuka A, Hara-Chikuma M (2012). Langerhans cells are critical in epicutaneous sensitization with protein antigen via thymic stromal lymphopoietin receptor signaling. J Allergy Clin Immunol.

[13] Sugita K, Steer CA, Martinez-Gonzalez I, Altunbulakli C, Morita H, Castro-Giner F (2018). Type 2 innate lymphoid cells disrupt bronchial epithelial barrier integrity by targeting tight junctions through IL-13 in asthmatic patients. J Allergy Clin Immunol.

[14] Drislane C, Irvine AD (2020). The role of filaggrin in atopic dermatitis and allergic disease. Ann Allergy Asthma Immunol.

[15] Akdis CA, Arkwright PD, Brüggen MC, Busse W, Gadina M, Guttman-Yassky E (2020). Type 2 immunity in the skin and lungs. Allergy.

[16] Jin H, He R, Oyoshi M, Geha RS (2009). Animal models of atopic dermatitis. J Invest Dermatol.

[17] Kim D, Kobayashi T, Nagao K (2019). Research techniques made simple: mouse models of atopic dermatitis. J Invest Dermatol.

[18] Yang C-C, Hung Y-L, Ko W-C, Tsai Y-J, Chang J-F, Liang C-W (2021). Effect of neferine on DNCB-induced atopic dermatitis in HaCaT cells and BALB/c mice. Int J Mol Sci.

[19] Mildner M, Jin J, Eckhart L, Kezic S, Gruber F, Barresi C (2010). Knockdown of filaggrin impairs diffusion barrier function and increases UV sensitivity in a human skin model. J Invest Dermatol.

[20] Pendaries V, Malaisse J, Pellerin L, Le Lamer M, Nachat R, Kezic S (2014). Knockdown of filaggrin in a three-dimensional reconstructed human epidermis impairs keratinocyte differentiation. J Invest Dermatol.

[21] Rouaud-Tinguely P, Boudier D, Marchand L, Barruche V, Bordes S, Coppin H (2015). From the morphological to the transcriptomic characterization of a compromised three-dimensional *in vitro* model mimicking atopic dermatitis. Br J Dermatol.

[22] Danso MO, van Drongelen V, Mulder A, van Esch J, Scott H, van Smeden J (2014). TNF-α and Th2 cytokines induce atopic dermatitis-like features on epidermal differentiation proteins and stratum corneum lipids in human skin equivalents. J Invest Dermatol.

[23] Kim D, Paggi JM, Park C, Bennett C, Salzberg SL (2019). Graph-based genome alignment and genotyping with HISAT2 and HISAT-genotype. Nat Biotechnol.

[24] Liao Y, Smyth GK, Shi W (2014). featureCounts: an efficient general purpose program for assigning sequence reads to genomic features. Bioinformatics.

[25] Perez-Riverol Y, Bai J, Bandla C, García-Seisdedos D, Hewapathirana S, Kamatchinathan S (2022). The PRIDE database resources in 2022: a hub for mass spectrometry-based proteomics evidences. Nucleic Acids Res.

[26] Jia T, Qiao W, Yao Q, Wu W, Kaku K (2019). Treatment with docosahexaenoic acid improves epidermal keratinocyte differentiation and ameliorates inflammation in human keratinocytes and reconstructed human epidermis models. Molecules.

[27] Ohtani T, Memezawa A, Okuyama R, Sayo T, Sugiyama Y, Inoue S (2009). Increased hyaluronan production and decreased E-cadherin expression by cytokine-stimulated keratinocytes lead to spongiosis formation. J Invest Dermatol.

[28] Tsoi LC, Rodriguez E, Degenhardt F, Baurecht H, Wehkamp U, Volks N (2019). Atopic dermatitis is an IL-13-dominant disease with greater molecular heterogeneity compared to psoriasis. J Invest Dermatol.

[29] Gittler JK, Shemer A, Suárez-Fariñas M, Fuentes-Duculan J, Gulewicz KJ, Wang CQF (2012). Progressive activation of T(H)2/T(H)22 cytokines and selective epidermal proteins characterizes acute and chronic atopic dermatitis. J Allergy Clin Immunol.

[30] Bogiatzi SI, Fernandez I, Bichet JC, Marloie-Provost MA, Volpe E, Sastre X (2007). Cutting edge: proinflammatory and Th2 cytokines synergize to induce thymic stromal lymphopoietin production by human skin keratinocytes. J Immunol.

[31] Kinoshita H, Takai T, Le TA, Kamijo S, Wang XL, Ushio H (2009). Cytokine milieu modulates release of thymic stromal lymphopoietin from human keratinocytes stimulated with double-stranded RNA. J Allergy Clin Immunol.

[32] Furue M (2020). Regulation of filaggrin, loricrin, and involucrin by IL-4, IL-13, IL-17A, IL-22, AHR, and NRF2: pathogenic implications in atopic dermatitis. Int J Mol Sci.

[33] Möbus L, Rodriguez E, Harder I, Stölzl D, Boraczynski N, Gerdes S (2021). Atopic dermatitis displays stable and dynamic skin transcriptome signatures. J Allergy Clin Immunol.

[34] Wang T, Zhang B, Li D, Qi X, Zhang C (2021). Bioinformatic analysis of key pathways and genes involved in pediatric atopic dermatitis. Biosci Rep.

[35] Vogel C, Marcotte EM (2012). Insights into the regulation of protein abundance from proteomic and transcriptomic analyses. Nat Rev Genet.

[36] Elias PM, Wakefield JS (2014). Mechanisms of abnormal lamellar body secretion and the dysfunctional skin barrier in patients with atopic dermatitis. J Allergy Clin Immunol.

[37] Gschwandtner M, Mildner M, Mlitz V, Gruber F, Eckhart L, Werfel T (2013). Histamine suppresses epidermal keratinocyte differentiation and impairs skin barrier function in a human skin model. Allergy.

[38] Aoshima M, Phadungsaksawasdi P, Nakazawa S, Iwasaki M, Sakabe J-I, Umayahara T (2019). Decreased expression of suprabasin induces aberrant differentiation and apoptosis of epidermal keratinocytes: possible role for atopic dermatitis. J Dermatol Sci.

[39] Beck LA, Cork MJ, Amagai M, De Benedetto A, Kabashima K, Hamilton JD (2022). Type 2 inflammation contributes to skin barrier dysfunction in atopic dermatitis. JID Innov.

[40] Leman G, Pavel P, Hermann M, Crumrine D, Elias PM, Minzaghi D (2022). Mitochondrial activity is upregulated in nonlesional atopic dermatitis and amenable to therapeutic intervention. J Invest Dermatol.

[41] Schmuth M, Jiang YJ, Dubrac S, Elias PM, Feingold KR (2008). Thematic review series: skin lipids. Peroxisome proliferator-activated receptors and liver X receptors in epidermal biology. J Lipid Res.

[42] Zhang W, Sakai T, Fujiwara S, Hatano Y (2017). Wy14643, an agonist for PPARα, downregulates expression of TARC and RANTES in cultured human keratinocytes. Exp Dermatol.

[43] Wopfner N, Dissertori O, Ferreira F, Lackner P (2007). Calcium-binding proteins and their role in allergic diseases. Immunol Allergy Clin North Am.

[44] Pfisterer K, Shaw LE, Symmank D, Weninger W (2021). The extracellular matrix in skin inflammation and infection. Front Cell Dev Biol.

[45] Bao L, Shi VY, Chan LS (2013). IL-4 up-regulates epidermal chemotactic, angiogenic, and pro-inflammatory genes and down-regulates antimicrobial genes *in vivo* and *in vitro*: relevant in the pathogenesis of atopic dermatitis. Cytokine.

[46] Chen X, Zhang Y, Pei J, Zeng X, Yang Y, Zhang Y (2022). Phellopterin alleviates atopic dermatitis-like inflammation and suppresses IL-4-induced STAT3 activation in keratinocytes. Int Immunopharmacol.

[47] Ulzii D, Kido-Nakahara M, Nakahara T, Tsuji G, Furue K, Hashimoto-Hachiya A (2019). Scratching counteracts IL-13 signaling by upregulating the decoy receptor IL-13Rα2 in keratinocytes. Int J Mol Sci.

[48] Egawa G, Kabashima K (2018). Barrier dysfunction in the skin allergy. Allergol Int.

[49] Kelsell DP, Byrne C (2011). SNPing at the epidermal barrier. J Invest Dermatol.

[50] de Koning HD, Rodijk-Olthuis D, van Vlijmen-Willems IM, Joosten LA, Netea MG, Schalkwijk J (2010). A comprehensive analysis of pattern recognition receptors in normal and inflamed human epidermis: upregulation of dectin-1 in psoriasis. J Invest Dermatol.

[51] Totsuka A, Omori-Miyake M, Kawashima M, Yagi J, Tsunemi Y (2017). Expression of keratin 1, keratin 10, desmoglein 1 and desmocollin 1 in the epidermis: possible downregulation by interleukin-4 and interleukin-13 in atopic dermatitis. Eur J Dermatol.

[52] Jung K, Imhof BA, Linse R, Wollina U, Neumann C (1997). Adhesion molecules in atopic dermatitis: upregulation of alpha6 integrin expression in spontaneous lesional skin as well as in atopen, antigen and irritative induced patch test reactions. Int Arch Allergy Immunol.

[53] Reynolds G, Vegh P, Fletcher J, Poyner EFM, Stephenson E, Goh I (2021). Developmental cell programs are co-opted in inflammatory skin disease. Science.

[54] Löffek S, Hurskainen T, Jackow J, Sigloch FC, Schilling O, Tasanen K (2014). Transmembrane collagen XVII modulates integrin dependent keratinocyte migration via PI3K/Rac1 signaling. PLoS One.

